# 100 DAYS OF COVID-19: RISK FACTORS AND CONFIRMED CASES IN 19 AFRICAN COUNTRIES

**DOI:** 10.21010/ajid.v15i2.6

**Published:** 2021-03-18

**Authors:** Philemon Dauda Shallie, Firoza Haffejee

**Affiliations:** 1,2 Department of Basic Medical Sciences, Durban University of Technology, KwaZulu-Natal, South Africa; 2 Department of Anatomy, Olabisi Onabanjo University, Sagamu Campus, Ogun State. Nigeria

**Keywords:** COVID-19, Risk factors, African Countries, Confirmed cases

## Abstract

**Background:**

The trail of the transmission of COVID-19 in Africa needs to be understood and conceptualized. With the limited response time to curb the transmission, the pandemic is already in 52 countries in Africa. There is much anxiety about the devastating potential of this scourge in Africa, justifiably so because of the weak health systems, high levels of poverty, and overcrowded cities. Therefore, this report examined the association between the confirmed cases at 100 days of COVID-19 and some significant risk factors in 19 African countries that had at least 100 confirmed cases as of 09 April 2020.

**Materials and Methods:**

We evaluated four major risk factors associated with COVID-19 confirmed cases in 19 African counties with over 100 cases in 100 days after the official declaration of COVID-19 by WHO.

**Results:**

Three of the four risk factors (total population in urban areas, population age, and international exposure) correlated positively with the number of COVID-19 cases. In contrast, one (public health system) correlated negatively with the number of confirmed cases in the countries under study. International exposure was initially the main transmitter of the infection, but community transmission now becomes the driver of COVID-19 infections on the continent.

**Conclusion:**

Identification of confirmed cases, quick contact tracing with self-isolation, community engagement, and health systems measures are all-necessary to prevent the potentially harmful ramifications of an epidemic on the continent. There is, therefore, the need for a comprehensive and integrated approach between the government and society.

## Introduction

The Chinese authority disclosed a large number of cases of pneumonia in Wuhan City, China, on 31 December 2019 (WHO, 2020a) and Thursday 09 April 2020 marked 100 days since the World Health Organisation (WHO) was officially informed about the severe acute respiratory syndrome coronavirus 2 (SARS-CoV-2) that caused the disease. The disease is now officially known as corona virus disease 2019 (COVID-19) (WHO, 2020a). The infection was relatively slow to reach sub-Saharan Africa, even as it spread rapidly across the world. The first case in Sub-Saharan African was reported in Nigeria on the 27th February 2020 and until mid-March, the virus was confined to less than a dozen countries which included Nigeria, Algeria, Egypt, South Africa, Ghana and Democratic Republic of the Congo (Sub-Saharan-Africa, 2020). Several Governments across the region took advantage of this delay. Angola began to enforce quarantine measures late in February for all people entering the country from high-risk destinations and it closed all its borders on 20 March, despite only recording its first two confirmed cases on 21 March. Notwithstanding having any confirmed cases as of 9^th^ of April, Lesotho, São Tomé and Príncipe and Comoros had closed their borders to all but their respective citizens and emergency services. As of 30 March 2020, 49 states in Africa had introduced limited or complete closures of their borders; closing airports, ports and in some cases land borders. By the same period, 44 sub–Saharan African countries had shut down schools, banned public congregations or put in place other social distancing measures and 11 had announced a state of emergency. Sub-Saharan Africa, as a region, arguably responded more quickly and decisively than anywhere else in the world (Sub-Saharan-Africa, 2020).

Although reported infection numbers in Africa were relatively small as of the 100 days, they are increasing rapidly, and the disease is projected to be ten times more deadly than the typical seasonal influenza, with reports of over 1.5 million infected people and nearly 80,000 deaths worldwide as of 9 April 2020 (Clarke Seán, 2020; Fausat and Del-Rio, 2020; WHO, 2020b).

The number of confirmed cases has remained very low in many African countries. This small number is cause for optimism, with the caveat that a lack of testing or transparent reporting may be significantly distorting these numbers (Sub-Saharan-Africa, 2020).

The trail of the transmission of COVID-19 in Africa needs to be understood and conceptualized. The limited knowledge has generated much anxiety about the devastating potential of this scourge in Africa, justifiably so because of the weak health systems, high levels of poverty and overcrowded cities. However, there is a ray of optimism that Africa will be able to avoid the worst of the pandemic considering the continent’s warmer climate, youthful population and experience in fighting infectious disease (Africa-Center-for-Strategic-Studies, 2020). Therefore, this report examined the association between the confirmed cases at 100 days of COVID-19 and the major risk factors for infection in 19 African countries that had at least 100 confirmed cases as of 9th April 2020. These risk factors include international exposure, population age, public health systems, the total population in urban areas and the population density of urban areas.

## Materials and Methods

To be able to respond appropriately to the pandemic, there is a need to understand the relative risks that each country faces. Five major risk factors (The population density of urban areas, the population of urban areas, International exposure, the public health system and the population age) were assessed. The relative level of each country’s vulnerability has been scaled (from 1 to 5, with 5 being greatest level of vulnerability and 1 the lowest). The data were sourced from the Africa Center for Strategic Studies, European Commission’s Joint Research Centre (EC JRC, 2020); Center for International Earth Science Information Network (CIESIN), 2020; World Bank, World exports, World development indicators and World Factbook. The data were used to construct the table while the regression analysis was determined using Pearson’s correlation coefficient.

Each of the variables was categorised on a scale of 1 – 5 as stated below:

1. The population density of urban areas is the measured population per square kilometre and was classified into five groups, with 5 being the highest density and 1 the lowest density (EC JRC, 2020; CIESIN, 2020). The scales are as follows:
  1. 0---25 persons/km^2^
  2. 25---75 persons/km^2^
  3. 75---150 persons/km^2^
  4. 150---300 persons/km^2^
  5. 300---500 persons/km^2^
2. The population of urban areas is determined as the total number of people living in urban areas as a percentage of the total population and classified into five groups;
  1. 20%
  2. 40%
  3. 60%
  4. 80%
  5. 100%.
  Hence 5 indicates the largest urban population and 1 the lowest urban population (EC JRC, 2020; CIESIN, 2020)
3. International exposure is a measure of travel, trade, tourism, or business with other countries (World Bank, 2020). These indices are aggregated and categorised according to the amount of spending in US dollars. With 5 being the greatest amount of spending and hence the greatest exposure, while 1 represents the least amount of spending and hence the least exposure. The categories are as follows:
  1. Less than $20 billion
  2. $20billion--$99 billion
  3. $100billion--$499 billion
  4. $500billion--$1 trillion
  5. More than $1 trillion.
4. The public health system was assessed based on the following weights to construct the overall composite measure: 25% for health; disability adjusted life expectancy (DALE), 25% for health inequality, 12.5% for the level of responsiveness, 12.5% for the distribution of responsiveness, and 25% for fairness in financing. These are categorised into five groups:
  1. 100%
  2. 80%
  3. 60%
  4. 40%
  5. 20%
  With 5 being the weakest and 1 being the strongest (world development indicators, 2020).
5. The World population age is usually categorised into five groups:
  0---14 years
  15—24 years
  25---54 years
  55---64 years
  65 years and above

The population age as a risk factor was determined from the percentage dependency age of those above the age of 65 years as a proportion of the total population. The percentage of each category varies from country to country, with 5 being the older population and 1 the younger population (The World Factbook, 2020).

## Results and Discussion.

**Table 1:**
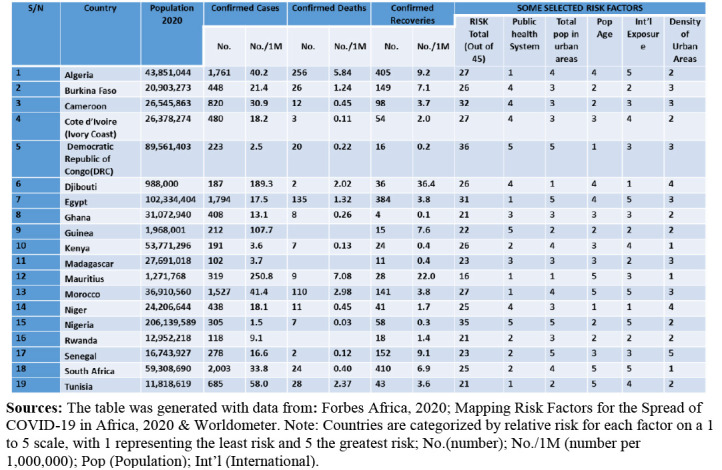
Selected Risk Factors and COVID-19 Statistics in selected African Countries at 100 Days of COVID-19.

**Figure 1:**
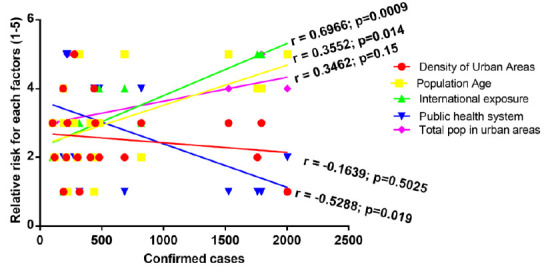
XY scatter plots of the relative risk factors and confirmed cases in some African Countries. The degree and nature of the correlation is given by the value of r (Pearson’s correlation coefficient). A value of p < 0.05 was considered statistically significant.

In this report, we evaluated five major risk factors associated with confirmed cases of COVID-19 in 19 African counties, each with a minimum of 100 cases, 100 days after the official declaration of COVID-19 by the WHO. There was a significant positive correlation of the number of cases of COVID-19 with international exposure (r = 0.6966; p = 0.0009) and population age (r = 0.3552; p = 0.014). The total population in urban areas was also positively correlated with the number of cases; however, this was insignificant (r = 0.3462; p = 0.15). The number of confirmed COVID-19 cases correlated negatively with public health systems (r = -0.5228; p = 0.019) and there was no correlation with population density of urban areas (r = -0.1639; p = 0.5052).

International exposure posed the highest risk because, except for China, the primary source of COVID-19 in all other countries was via international exposure, a possible reason for the epidemic starting late in Africa (Martinez-Alvarez Melisa, Brotherton Helen, Antonio Martin, & Anna, 2020). Nevertheless, African countries such as Algeria, Egypt, Morocco, South Africa and Nigeria, who have the highest level of international exposure, were among the earliest to be affected. However, as of 100 days since the official declaration of the pandemic, COVID-19 had already infected people in 52 African countries. Consequently, community transmission is the secondary source of new infections.

In addition to international exposure, population age is the second predisposing factor; 80% of deaths, in the USA, from the coronavirus have been in people 65 years and older. About 6% to 29% of people over the age of 85 years who are infected with COVID-19 will require intensive care because of their weaker immune systems predisposing them to infections and more likely to have long-term health problems that put them at risk (Maragakis, 2020). The African countries with high risk of age as a predisposing factor include South Africa with 5.3%, Morocco with 6.8%, Tunisia with 8.05% and Mauritius with 6.8% of their respective populations, above 65 years old.

The total population depicts the population at higher risk for infections. Furthermore, overpopulation is a major factor in the transmission of COVID-19; viruses thrive and spread faster in densely populated areas, at close range through movement and frequent contact between people (Nakazato and Takano, 2020). Although our result on population density appeared contrary to this in the early stage of the infection, it will nevertheless be the major factor in community transmission, with the progress of time. High-risk countries include South Africa, Senegal, Nigeria, Morocco, Kenya, Egypt, the Democratic Republic of Congo, and Algeria. Some of these countries have large urban populations in megacities like Johannesburg and Lagos, which in turn have the most substantial infections in South Africa and Nigeria, respectively. In most of these large cities, people from the lower socio-economic strata live in townships and informal settlements, where residents lack the financial means to stock up food and other necessities. The inadequate shelter, particularly in informal settlements, also makes the enforcement to stay at home difficult. This, coupled with the overcrowded living conditions, would make it difficult for infected people to self-isolate and pose difficulties of contact tracing and difficulties to isolate people who have been exposed to an infected person.

Although the healthcare systems thus far correlated negatively with the number of confirmed cases, this may, however, play a pivotal role going forward in stemming the tide of infections and facilitating recoveries. Unfortunately, healthcare systems in most of the sub-Saharan countries are very poor, due to meagre public health spending in sub-Saharan Africa at an average of 5.2% of gross domestic product (GDP), compared with 10% in other countries globally (Sub-Saharan-Africa, 2020).

## Conclusion:

The initial outbreak of the epidemic in Africa was through international exposure and those African countries with the highest international exposure had the highest number of cases of COVID-19. However, as countries have now closed their borders, other risk factors such as the age of the population have a direct impact on transmission. The health systems of a country are negatively associated with transmission. In this early stage of the disease, population numbers and density in urban areas did not play a role but would be important risk factors as the disease progresses in time. Identification of confirmed cases, quick contact tracing with self-isolation, community engagement and health systems measures are all necessary to prevent the potentially harmful ramifications of an epidemic on the continent. There is, therefore, the need for a comprehensive and integrated approach between the government and society.

**Abbreviations:**
WHO: - World Health Organisation
SARS-CoV-2: - Syndrome Coronavirus 2
COVID-19: - Corona-Virus Disease 2019
EC JRC: - European Commission’s Joint Research Centre
CIESIN: - Centre for International Earth Science Information Network
GDP: - gross domestic product
